# Atf3 Promotes Spinal Cord Injury by Exacerbating Neuronal Oxidative Stress and Inflammation via the NF-*κ*B Signaling Pathway

**DOI:** 10.1155/ijog/1027388

**Published:** 2025-08-11

**Authors:** Haijian Yan, Pingjiang An, Jingjing He, Bin Zhang, Binxing Wei, Tongqian Wu, Qing Li

**Affiliations:** ^1^Department of Emergency, Affiliated Hospital of Guizhou Medical University, Guiyang, China; ^2^School of Clinical Medicine, Guizhou Medical University, Guiyang, China; ^3^Department of Clinical Laboratory, Guizhou Hospital, The First Affiliated Hospital of Sun Yat-sen University, Guiyang, China; ^4^Clinical Research Center, Affiliated Hospital of Guizhou Medical University, Guiyang, China

**Keywords:** Atf3, inflammation, NF-*κ*B/p65, oxidative stress, spinal cord injury

## Abstract

**Background:** Spinal cord injury (SCI) is a central nervous system disorder characterized by oxidative stress and inflammatory responses. Activating transcription factor 3 (Atf3) is an early-response stress-regulating gene whose abnormal upregulation exacerbates oxidative stress and inflammation. However, despite its known association with oxidative stress, the mechanism of Atf3 in SCI remains incompletely understood. This study aimed to investigate the role and mechanisms of Atf3 in SCI.

**Methods:** Bioinformatics analysis was performed using multiple GEO datasets to identify hub genes and key signaling pathways associated with post-SCI oxidative stress. The rat SCI model and a lipopolysaccharide (LPS)-induced PC12 cell injury model were established in vivo and in vitro to investigate whether Atf3 could ameliorate SCI progression, neuronal damage, oxidative stress, and inflammation. Chromatin immunoprecipitation quantitative PCR (ChIP-qPCR) and luciferase reporter assays were used to analyze the interaction between Atf3 and NF-*κ*B/p65, as well as the promoter activity of the p65 gene. Finally, the NF-*κ*B signaling pathway was activated to observe the role of Atf3 in neuronal injury and SCI.

**Results:** Bioinformatics analysis revealed significant enrichment of Atf3 and the NF-*κ*B signaling pathway in SCI datasets. Atf3 primarily colocalized with NeuN but minimally with GFAP or Iba-1. Knockdown of Atf3 significantly alleviated SCI damage, reduced oxidative stress (decreased MDA/MPO and increased SOD/GSH levels), and suppressed inflammation (reduced TNF-*α*, IL-1*β* and IL-6 levels) in both SCI rats and LPS-treated PC12 cells. However, activation of NF-*κ*B counteracted these protective effects. ChIP-qPCR and luciferase reporter assays demonstrated that Atf3 overexpression enhanced its binding to the p65 promoter and promoted NF-*κ*B/p65 activation, whereas Atf3 knockdown reversed these effects.

**Conclusion:** Downregulation of Atf3 mitigates oxidative stress and inflammation in SCI, potentially by regulating neuronal biological functions via the NF-*κ*B pathway. These findings provide a theoretical foundation for understanding SCI pathogenesis and identifying therapeutic targets.

## 1. Introduction

SCI is a traumatic central nervous system (CNS) injury consisting of primary and secondary injury phases. Primary injury leads to irreversible cellular dysfunction and death, thereby triggering a cascade of secondary injury events including ischemic axonal damage, neuronal apoptosis/necrosis, microvascular disruption, and inflammatory responses [1]. Secondary injury develops within hours to weeks, with edema, neuroinflammation, ischemia, and dysregulated cytokine and oxidative stress (OS) responses that promote further neuronal loss [2]. These processes worsen spinal cord damage and hinder recovery. Therefore, understanding secondary injury mechanisms is crucial for developing therapies to limit neural damage and improve outcomes [3].

OS and neuroinflammation are central to secondary SCI pathogenesis [4]. Redox imbalance after SCI generates excess reactive oxygen species (ROS), damaging nucleic acids, lipids, and proteins. ROS-induced mitochondrial dysfunction further increases ROS production and triggers inflammation, worsening neuronal injury [5, 6]. Key pathways such as NF-*κ*B and Nrf2 are activated during OS, driving proinflammatory cytokine release from microglia and macrophages. These cytokines disrupt the blood-spinal cord barrier, activate astrocytes, and perpetuate neuroinflammation, ultimately accelerating neuronal death [7]. Recent studies highlight the central role of OS and inflammation in SCI progression [8, 9].

Activating Transcription Factor 3 (Atf3) is a key regulator of cellular stress responses within the ATF/CREB family. It controls signaling pathways related to apoptosis, ferroptosis, and differentiation [10]. Under homeostasis, Atf3 remains transcriptionally silent but is rapidly induced by stressors to modulate target gene networks and cellular adaptations [11]. As a regeneration-associated gene (RAG), Atf3 is upregulated in injured spinal neurons, promoting axonal regeneration and neuroprotection [12, 13]. Paradoxically, Atf3 also exacerbates OS and inflammation in cardiovascular disease models, reflecting its context-dependent duality [14]. In murine embryonic fibroblasts, Atf3 suppresses inflammation via NF-*κ*B inhibition or promotes M2 macrophage polarization by regulating CCL4, thereby attenuating hepatic inflammation [15, 16].

The NF-*κ*B pathway, a central regulatory hub in the CNS, modulates synaptic plasticity, memory formation, and neuroinflammatory amplification [17]. Post-SCI, astrocytic NF-*κ*B activation drives IL-6 production, which intensifies neuroinflammation and impairs recovery [18]. Bidirectional crosstalk between ROS and NF-*κ*B further complicates this interplay, with NF-*κ*B modulating intracellular ROS levels [19, 20]. Emerging evidence suggests Atf3 exerts anticancer effects on HPV16-associated cervical cancer cells by hindering cell growth and inducing cell cycle arrest through the NF-*κ*B signaling pathway [21]. Despite these advances, Atf3's regulatory mechanisms in SCI remain elusive.

Although the potential regulatory role of Atf3 in nerve injury has been suggested, its specific function in SCI pathogenesis remains unclear. In this study, we demonstrated that Atf3 may regulate the NF-*κ*B signaling pathway to influence neuronal OS and inflammatory responses during SCI progression. Our findings reveal the critical role of Atf3 in SCI and may provide new molecular targets for intervention strategies and a theoretical basis for the treatment of SCI.

## 2. Material and Methods

### 2.1. Bioinformatics Analysis

#### 2.1.1. Analysis of Differentially Expressed Genes (DEGs)

The GSE5296, GSE171441, and GSE47681 datasets were downloaded from the GEO database (https://www.ncbi.nlm.nih.gov/geo/). The GSE5296 dataset contains 28 samples from rats at 0.5 and 4 h and 1, 3, 7, and 28 days, and the sham-operated group after SCI. The GSE171441 dataset contains rats 3, 14, and 35 days after SCI and in the sham-operated group, totaling 16 samples. The GSE47681 dataset contains rats 1, 3, and 7 days after SCI and in the sham-operated group, totaling 16 samples. The transcriptome data were processed using the sva package (using the ComBat function) software package in R (http://www.datavis.ca/r/) to remove batch effects. The gene expression matrix of the GSE5296 microarray data was differentially analyzed by the Limma bioinformatics tool based on the R language (v4.3.1).

#### 2.1.2. Gene Ontology (GO) and Kyoto Encyclopedia of Genes and Genomes (KEGG) Enrichment Analysis and Gene Set Enrichment Analysis (GSEA)

The OS differential genes were subjected to GO enrichment and KEGG pathway enrichment to further understand the biological processes (BPs) in which the above gene sets may be involved. The GO analyses selected BP, cellular component (CC) and molecular function (MF) for GO and KEGG pathway analysis with a threshold of *p* < 0.05.

GSEA (http://www.gsea-msigdb.org/) ranked all genes analyzed in the expression arrays according to their expression differences between disease and control groups. GSEA calculates a normalized enrichment score for each gene set. Positive enrichment score values represent enrichment at the top of the list, and negative enrichment score values represent enrichment at the bottom of the list. Pathway maps of the GSEA enrichment analysis were then drawn to graphically represent the enriched biological pathways using R.

#### 2.1.3. Intersection Analysis of Weighted Gene Coexpression Network Analysis (WGCNA) and Oxidative Stress–Associated DEGs (OS-DEGs)

The GSE5296 normalized gene expression profiles were analyzed by the WGCNA toolkit in R (Version 4.3.1) to construct gene coexpression networks. We first computed pairwise Pearson correlation coefficients between genes to construct a gene coexpression similarity matrix. Subsequently, a soft thresholding power (*β* = 5) was applied to transform this similarity matrix into a weighted adjacency matrix, with the threshold value determined by achieving a scale-free topology fit index of *R*^2^ = 0.9. This approach ensured that the resultant network exhibited scale-free properties consistent with biological systems. The topological overlap matrix (TOM) algorithm was further used to optimize the network structure, and the minimum module size was set to 30 genes by dividing the functional modules through hierarchical clustering combined with the dynamic tree-cutting algorithm. Module eigengenes (MEs) were used as the core expression indexes of each module, and the intra-module contribution of genes and their correlation with phenotypes were assessed by calculating module membership (MM) and gene significance (GS), respectively. Finally, the significant module (*p* < 0.05, corrected by Bonferroni) was taken as the intersection with 82 OS-DEGs, and combined with the *t*-test (FDR < 0.05) to screen out the candidate genes with dual regulatory features.

### 2.2. Multiple Machine Learning Methods

To construct a machine learning framework for multidimensional feature screening, three complementary strategies were applied: LASSO regression, random forest (RF) ensemble learning, and support vector machine recursive feature elimination (SVM-RFE). LASSO employs L1 regularization to shrink coefficients of nonessential features to zero, achieving sparse feature selection. RF integrates bootstrap resampling and decision tree architectures, dynamically evaluating feature importance via the Gini index for robust prediction of continuous variables. SVM-RFE utilizes the maximum margin principle and a recursive backward elimination strategy to iteratively rank and optimize features. The three types of methods synergistically improve the generalization performance and biological explanatory power of predictive models from the dimensions of regularization constraints, integrated learning, and iterative optimization, respectively. The R environment (v4.3.1) was employed to process 82 OS-DEGs. First, the glmnet and caret packages were loaded for LASSO regression (*λ* = 1 SE), followed by the RF package for RF analysis (Top 10) and then the msvmRFE.R package for SVM-RFE analysis (Importance > 0.2). Each set of key genes was entered as a collection into the Venn diagramming tool to identify common genes between different collections. The identified overlapping genes—macrophage inflammatory protein 1*α* (Ccl3, C-C motif chemokine ligand 3) and Atf3—were validated across GSE5296, GSE171441, and GSE47681 datasets.

### 2.3. Rat Model

Fifty-four female Sprague–Dawley (SD) rats that were eight-week-old were purchased from the Beijing Vital River Laboratory (Beijing, China). Urethane (1.5 mg/kg) was administered before the surgery via an intraperitoneal injection. After successful anesthesia, the animals were fixed on the operating platform in the prone position. A longitudinal incision was made on the dorsal side of the T9-T10 vertebral body 2–3 cm. The paraspinal muscles were stripped and the spinous process and lamina of T9–T10 were removed to fully expose the spinal cord. With a closing force of 50 g, the aneurysm clip (Kent Scientific) was clipped epidurally for 1 min, and the lower limbs of the rats showed a retraction-like swing and flaring paralysis [22]. After removal, the skin was sutured to achieve hemostasis. The average operation time was 20 min. All rats were housed individually in a temperature-controlled room at 22 ± 3°C and provided water and food ad libitum. During this period, the bladders of the animals were manually voided twice daily until they returned to normal bladder function.

For therapeutic intervention, shAtf3 (8 *μ*L, 8.64 × 10^8^ TU/mL, RiboBio, China) was administered via intrathecal injection 2 days prior to the surgical procedure [23]. Phorbol 12-myristate 13-acetate (PMA) (20 ng/kg, Sigma P1585) was intraperitoneally injected after SCI to activate the NF-*κ*B signaling pathway. The animals were euthanized 24 h after SCI surgery, and the L2 segments of the spinal cord were harvested for further examination. Before and during the experiments, all rats were housed together for at least 1 week under specific pathogen-free conditions. All animal experiments were approved by the Animal Experiment Ethics Committee of Guizhou Medical University (No. 2305520) to ensure that the entire research process complied with ethical standards. Our experimental procedures were performed in compliance with the national standard of China: GB/T 35892-2018 Laboratory Animal - Guidelines for the Ethical Review of Animal Welfare, which aims at the protection and welfare of animals used in scientific research.

### 2.4. Cell Lines and Treatment

The PC12, a neuronal cell line, was sourced from Pricella (Wuhan, China). These cells were cultured in Iscove's Modified Dulbecco's medium (IMDM) supplemented with 10% fetal bovine serum (FBS; Gibco, United States) from Hyclone (United States) and 1% penicillin/streptomycin (Sigma, United States). Cells were maintained in an incubator set at 37°C in an atmosphere of 5% CO2.

The short hairpin RNA targeting Atf3 (shAtf3), Atf3 overexpression vectors, and their respective negative controls (siNC and NC) (14.32 *μ*L, 8.64 × 10^8^ TU/mL) were procured from RiboBio (RiboBio, China). Cells were transfected for 48 h using Lipofectamine 2000 (Thermo, United States) according to the manufacturer's protocol to ensure efficient delivery of genetic material with three lentiviral vectors harboring distinct interference target sequences (Supporting Information 1, Table S1). The transfection efficiency was verified by PCR to select the optimal target (Figure S1). The NF-*κ*B activator PMA (Sigma P1585) was administered to the cell culture medium at a dose of 2 *μ*M for 24 h. Cells were collected for subsequent experiments.

### 2.5. Quantitative PCR (qPCR)

Spinal cord tissue and PC12 cells were homogenized in TRIzol buffer (Thermo, United States). RNA (1 *μ*g) was reverse transcribed to cDNA using a First Strand Synthesis Kit (Transgen, China), following the manufacturer's protocol. Gene expression was measured using the SYBR Green mixture (Takara, Japan). GAPDH was used as an internal control, and the primers for relevant genes are shown in Table S2.

### 2.6. Histological Analysis

Spinal cord tissue was obtained from the L2 segment. The spinal cord tissues were isolated, washed with PBS, fixed in 4% PFA, dehydrated, embedded in paraffin, and sliced into 5 *μ*m sections. The samples were stained with hematoxylin and eosin (H&E; Beyotime, China), Masson's trichrome (Beyotime, China), and Nissl stain (Beyotime, China) to assess tissue damage, matrix integrity, and neuronal activity, respectively. Apoptosis in the tissues was measured using a TUNEL assay kit (Beyotime, China), according to the manufacturer's protocol. Images were obtained using a microscope (Leica). The localization of Atf3 in spinal cord tissue was measured by immunofluorescence staining with NeuN (neuronal marker), GFAP (astrocyte marker), and Iba-1 (microglial marker).

### 2.7. Enzyme-Linked Immunosorbent Assay (ELISA)

Spinal cord tissues were harvested and processed into a homogenate, and the cell culture supernatant was collected for assessment of inflammatory cytokines. The concentrations of IL-1*β*, IL-6, and TNF-*α* were measured using a commercial ELISA kit (Thermo, United States). ELISA was performed in accordance with the manufacturer's recommended protocols to ensure accurate quantification of these cytokines.

### 2.8. Western Blotting Assay

Total protein extraction from tissues and PC12 cells was performed using RIPA lysis buffer (Thermo, United States). Equal quantities of protein samples were resolved on an 8%–10% sodium dodecyl sulfate-polyacrylamide gel electrophoresis (SDS-PAGE) gel and then transferred onto polyvinylidene fluoride (PVDF) membranes (Millipore, United States). The membranes were blocked with 5% nonfat milk to prevent nonspecific binding.

Subsequently, membranes were incubated with specific primary antibodies (Abcam, United States) at 4°C overnight. Immunoreactive protein bands were analyzed by incubating the membranes with horseradish peroxidase (HRP)-conjugated secondary antibodies (Thermo, United States) specific to either rabbit or mouse immunoglobulins. After the addition of an ECL reagent from Millipore, the protein bands were visualized using a gel image analysis system, allowing for documentation and quantification of the immunoblots.

### 2.9. Cell Viability

Cell viability was assessed using the Cell Counting Kit-8 (CCK-8, Beyotime, China), following the manufacturer's protocol. Cells were seeded into a 96-well plate at a density of 5,000 cells per well and treated with LPS or PBS as a control. Following a 24-h incubation period, the CCK-8 reagent was added to each well and allowed to react for 2 h. The absorbance of the wells was measured at 450 nm using a microplate reader to determine the cell viability.

### 2.10. Chromatin Immunoprecipitation (ChIP) Assay

The interaction between Atf3 and p65 was detected using the ChIP assay. According to the manufacturer's instructions of the EZ-ChIP kit (Millipore, United States), cells were transfected with shAtf3 or Atf3 overexpression vectors for 24 h, then fixed with formaldehyde for 10 min to obtain DNA-protein crosslinks. Cell lysates were sonicated to generate chromatin fragments of 200–300 bp and immunoprecipitated with Atf3-specific antibodies (Thermo, United States) or IgG (Thermo, United States) as a control. The precipitated chromatin DNA was analyzed by qPCR to determine p65 levels (Supporting Information 2; Figures S1 and S2).

### 2.11. Luciferase Reporter Gene Assay

Luciferase reporter gene vectors containing wild-type (WT) or mutant (MUT) sequences of the p65 promoter region were inserted into the pGL3-basic vector. PC12 cells were cotransfected with these vectors and shAtf3 or Atf3 overexpression vectors. Luciferase activity was measured using a dual-luciferase reporter gene assay system (Promega, United States).

### 2.12. Cell Apoptosis

Cell apoptosis was measured by flow cytometry to determine the profile of Annexin V (BD Biosciences, Franklin Lakes, New Jersey, United States) and propidium iodide (PI) (BD Biosciences) or colorimetric TUNEL apoptosis assay kit (Bioyotime, Shanghai, China). In brief, 2.0 × 10^5^ sh-NC or sh-Atf3 PC12 cells were incubated with LPS for 24 h. To investigate the role of NF-*κ*B, cells will be treated with LPS or PMA for 24 h. Next, cells were stained with APC-annexin V/PI for 20 min followed by analysis by a flow cytometer (Beckman coulter, Navios). For the TUNEL assay, the cells were fixed with 4% paraformaldehyde for 30 min, permeabilized with 0.3% Triton X-100, blocked with 0.3% H2O2, and then incubated with TUNEL assay solution at 37°C for 60 min and observed by fluorescence microscopy. The TUNEL-positive ratio was quantified using ImageJ software.

### 2.13. Immunofluorescence Staining

After the tissues were dewaxed and rehydrated by gradient ethanol and xylene, the antigens were thermally repaired, and the nonspecific sites were blocked with 5% BSA (containing 0.3% Triton X-100, Beyotime, China). The tissues were sequentially incubated with primary antibodies diluted proportionally, including anti-Atf3 (Thermo, United States), anti-NeuN, anti-GFAP, anti-Iba-1 (all from Abcam, USA), and anti-Caspase-3 (Santa Cruz, United States) overnight at 4°C. Fluorescent secondary antibodies Goat Anti-Rabbit IgG H&L (Alexa Fluor 488) (Thermo, United States) or Goat Anti-Rabbit IgG H&L (Alexa Fluor 594) at 37°C for 1 h were used and washed with PBS several times, and then, the nuclei were stained with DAPI (Beyotime, China). The slices were blocked with antiquencher, and the target proteins and nuclei localization were observed by fluorescence microscopy. The fluorescence intensity of the target protein was quantified using ImageJ software.

### 2.14. Statistical Analysis

Data were analyzed using GraphPad Prism 9.0. Normally distributed data were expressed as mean ± SD and analyzed by one-way ANOVA with the least significant difference (LSD) test used for post hoc comparisons. Nonnormally distributed data were analyzed using the Kruskal–Wallis *H*-test. For time-course data, a repeated measures ANOVA was performed. Sample sizes were determined using the resource equation approach, considering the exploratory nature of the study and challenges in estimating standard deviation and effect size. *p* < 0.05 was considered statistically significant.

## 3. Results

### 3.1. DEG Analysis

**Figure 1:**
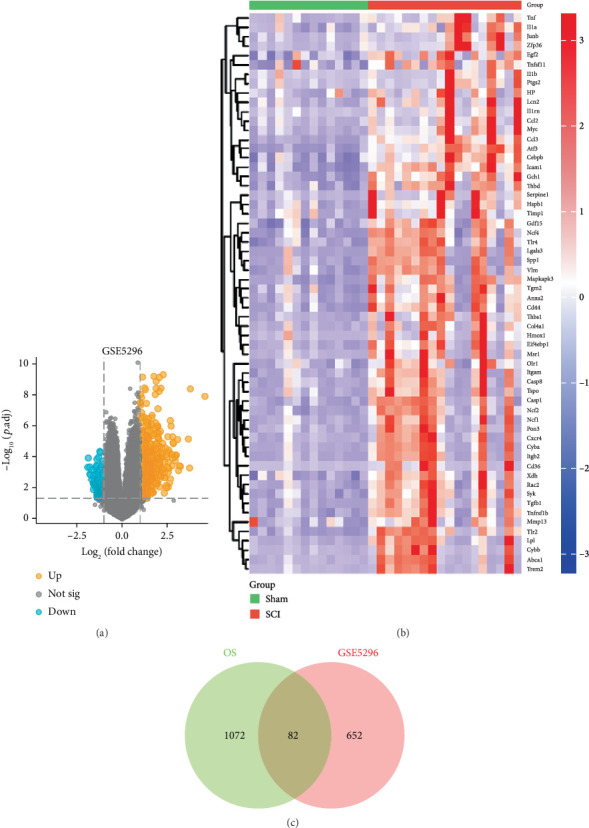
Differentially Expressed Genes of SCI and oxidative stress were obtained. (a) Volcano plot of SCI-related DEGs in GSE5296. DEGs marked in orange are up-regulated genes, blue corresponds to down-regulated genes, while grey represents genes that are not meeting the significance threshold. (b) Heatmap of DEGs in GSE5296. Red indicates genes whose expression is significantly up-regulated in the SCI group, blue indicates genes that are significantly down-regulated, and the intensity of the color block is positively correlated with the normalized expression values. (c) Venn diagram showing overlap between SCI-associated DEGs and oxidative stress-related genes.

In this study, DEGs are the genes with statistically significant differences in gene expression levels between rats with spinal cord injury and those in the sham operation group, reflecting the genetic changes during the major process of spinal cord injury [24]. DEGs were visualized by a coordinate system with the horizontal (*X*) axis indicating log2-fold change and the vertical (*Y*) axis indicating −log10 adjusted *p* value, and by a continuous color gradient indicating high or low gene expression. Screening of DEGs was performed according to the two significance threshold criteria (|log2FC| ≥ 1 as the threshold and *p* < 0.05) (Figure 1a,b). The OS gene set was further obtained from GeneCards (Keywords: Oxidative stress, relevance score > 5), and the intersection of these DEGs and OS genes was taken to obtain 82 OS-DEGs (Figure 1c), and the multidimensional data integration strategy was used to finally locate the differentially regulated genes that are closely related to the pathological process of OS.

### 3.2. GO, KEGG Enrichment Analysis, and GSEA for OS-DEGs

**Figure 2:**
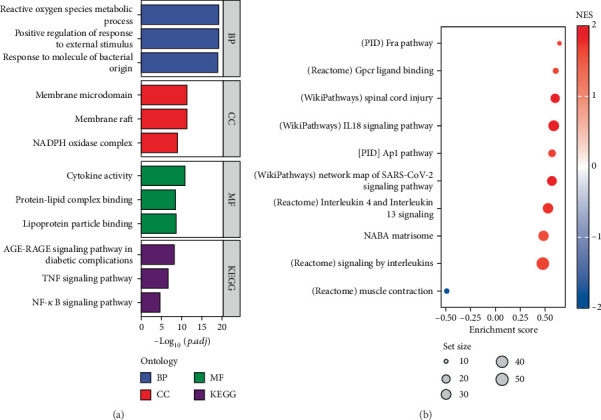
Oxidative stress DEGs were analyzed by GO, KEGG, and GSEA. (a) Enrichment analysis of genes positively associated with oxidative stress: BP, cellular component (CC), MF, and KEGG pathway. (b) GSEA of oxidative stress-associated genes.

GO systematically describes the basic MF, BP, and cellular localization of genes through structured terminology, while KEGG focuses on the networked mechanisms of genes in advanced biological pathways such as metabolism and signal transduction [25]. The results of GO analysis suggested that the enrichment of DEGs is important in the BPs such as cytokine activity and ROS metabolism, while KEGG analysis suggested that the NF-*κ*B signaling pathway and TNF signaling pathway were significantly enriched (Figure 2), suggesting that they may play an important role in the OS process. The GSEA analysis suggests that the DEGs in GSE5296 are significantly enriched in multiple signaling pathways and BPs that are closely related to OS (Figure 2).

### 3.3. Intersection Analysis of WGCNA and OS-DEGs

**Figure 3:**
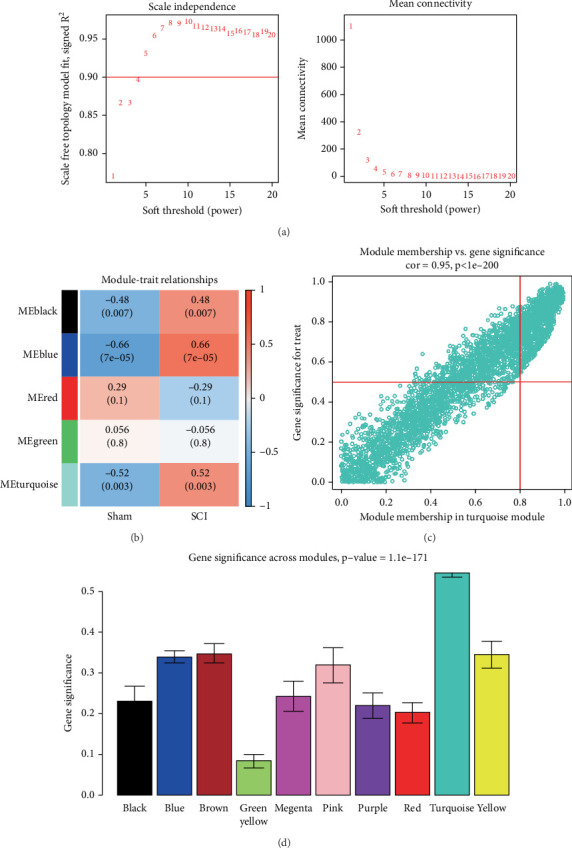
WGCNA was used to determine the correlation between genes to reveal functional association. (a) Selection of optimal soft threshold power (*β* = 5, *R*^2^ = 0.9). (b) Module–trait relationship heatmap. (c) Scatter plot between GS and MM in the turquoise module. The trend line shows a positive correlation. (d) Box plot of GS distribution.

We performed WGCNA to identify correlations between genes and construct a network, clustering genes with similar expression patterns into modules to uncover functional associations. Pearson correlation coefficients were calculated to establish an initial similarity matrix, followed by the application of a soft thresholding power (*β* = 5; scale-free topology fitting index *R*^2^ = 0.9) to generate a weighted adjacency matrix (Figure 3a). Subsequent correlation analysis with clinical traits identified five phenotype-associated gene modules (Figure 3b). The gene clusters in the turquoise coexpression module exhibited significant positive correlations in the SCI model, suggesting that there is a significant trend of synergistic changes in the gene expression pattern of this module with the pathological process of SCI (Figure 3c). Taking the intersection of the turquoise module genes with the 82 OS-DEGs, 82 OS-DEGs were found to be contained within the turquoise module gene set. The turquoise module is significantly higher than the other modules (Figure 3d).

### 3.4. Multiple Machine Learning Methods Were Used to Screen Hub Genes

**Figure 4:**
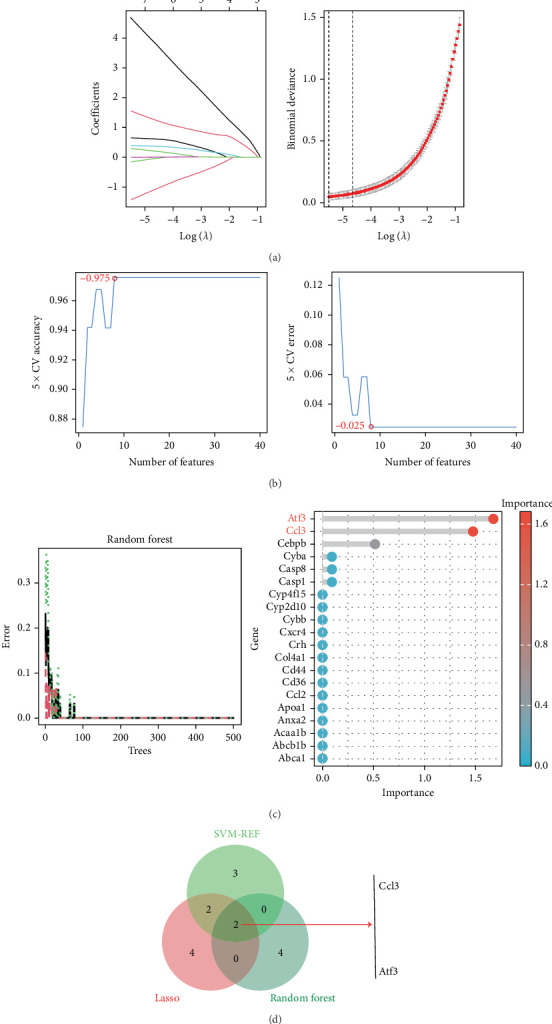
Hub gene was screened using a variety of machine learning methods. (a) The LASSO algorithm was used to determine the optimal penalty coefficient *λ* through cross-validation, and 8 genes with nonzero coefficients were selected as the robust feature set. (b) Based on the SVM-RFE algorithm, the radial basis kernel function was used to rank the feature importance, and the optimal subset of 7 key genes was obtained. (c) After feature recursive segmentation and optimization by the random forest algorithm combined with the classification decision tree, six key genes with high confidence were identified. (d) Through the integrated analysis of the feature weight matrices of the three machine learning algorithms and the three-dimensional Venn diagram visualization, the system identified two core hub genes with cross-algorithm consistency.

By using machine learning methods such as LASSO regression, SVM-RFE, and RF, LASSO regression analysis was first performed to obtain eight genes (Figure 4a). Subsequently, the SVM-RFE algorithm was performed to obtain seven genes (Figure 4b). Finally, the RF algorithm was used to obtain six genes (Figure 4c). Based on the multialgorithm cross-validation strategy, combining the screening results of the above three algorithms and using Venn diagram analysis for cross-algorithm consistency, the core candidate genes with cross-algorithmic consistency were systematically identified as Ccl3 and Atf3 (Figure 4d).

### 3.5. Expression and Validation of Hub Genes

**Figure 5:**
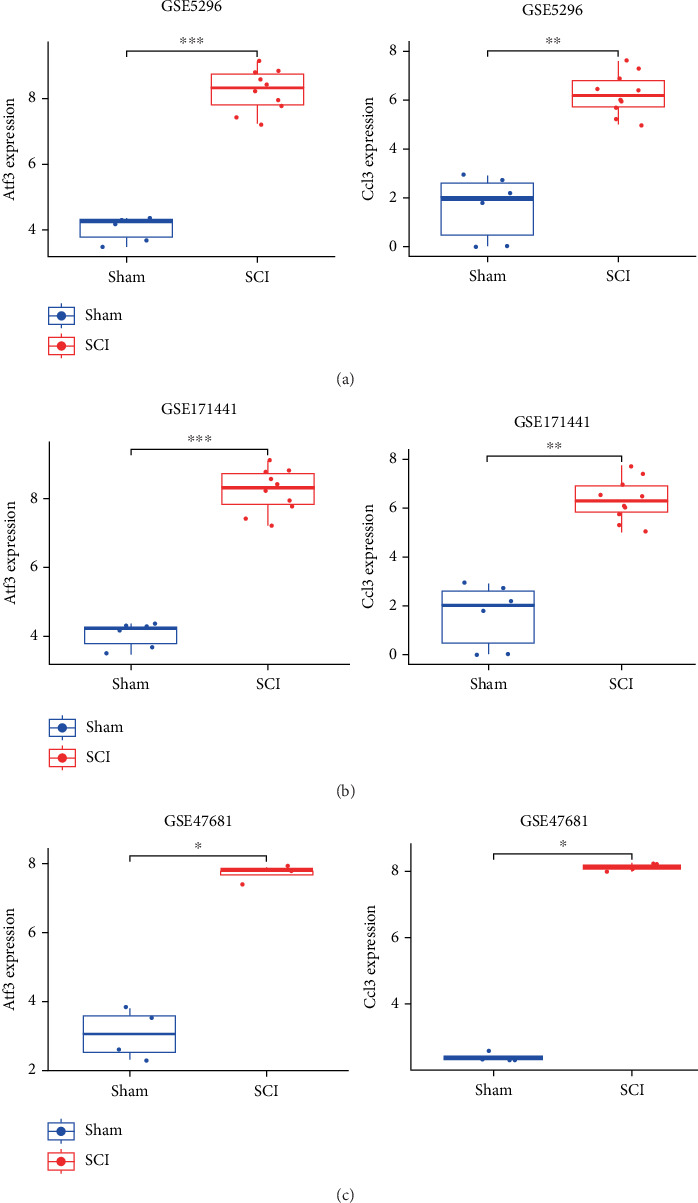
Hub genes (Ccl3 and Atf3) were validated in GSE5296, GSE171441, and GSE47681 datasets. (a) Expression of Ccl3 and Atf3 in the GSE5296 dataset. (b) Atf3 expression in the GSE171441 dataset (SCI time course: 3/14/35 days). (c) Ccl3 and Atf3 expression in the GSE47681 dataset (FDR < 0.01, one-way ANOVA). (∗*p* < 0.05, ∗∗*p* < 0.01, ∗∗∗*p* < 0.001).

The Ccl3 and Atf3 were validated across GSE5296, GSE171441, and GSE47681 datasets. Boxplot analysis revealed that Atf3 expression was significantly upregulated (*p* < 0.05, Student's *t*-test) in SCI groups compared to sham controls across all three datasets (Figures 5a, 5b, and 5c). Based on this multidataset validation, Atf3 was selected as the hub gene associated with OS in spinal cord injury.

### 3.6. Knockdown of Atf3 Alleviates Spinal Cord Injury, OS, and Inflammatory Response In Vivo

**Figure 6:**
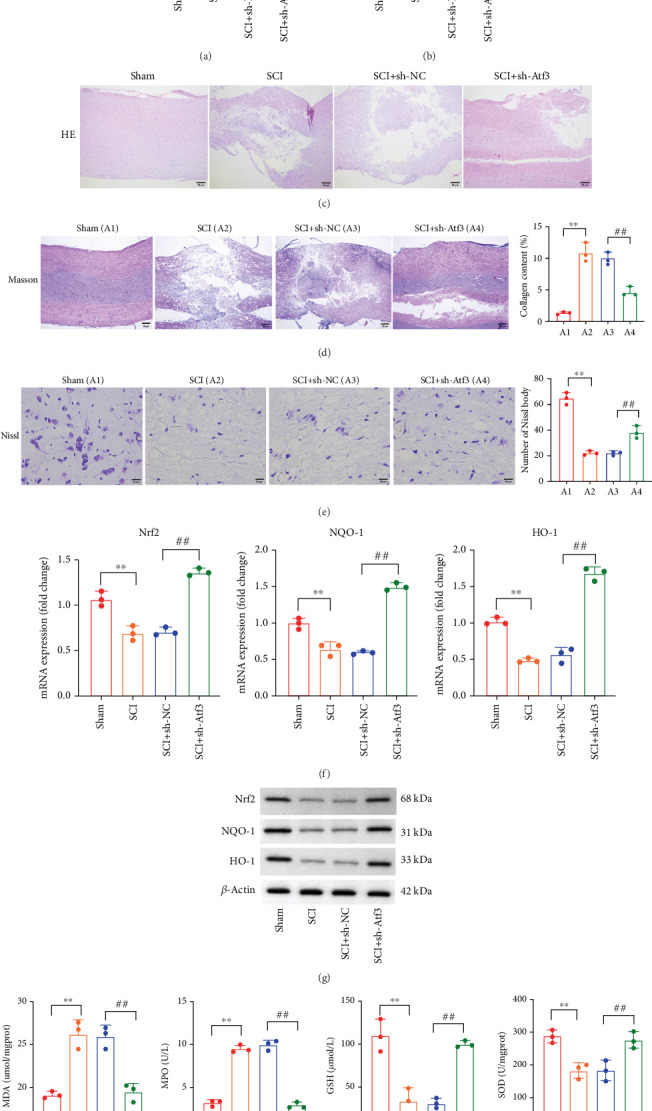
Knockdown of Atf3 attenuates SCI and oxidative stress. The (a) mRNA expression level and (b) protein expression levels of Atf3 in spinal cord tissues of Sham, SCI, SCI+sh-NC, and SCI + sh-Atf3 groups were analyzed by qPCR and Western blot. (c) HE staining, (d) Masson staining, and (e) Nissl staining of spinal cord tissues from each group (scale bar = 50* μ*m). (f) mRNA and (g) protein levels of antioxidant enzymes (Nrf2, NQO-1 and HO-1) in spinal cord tissues of each group were analyzed by qPCR and Western blot. (h) Expression levels of oxidative enzymes (MDA and MPO) and antioxidant enzymes (GSH and SOD) in spinal cord tissues of each group. Data are presented as mean ± SD, *n* = 3. ∗∗*p* < 0.01 versus Sham group; ^##^*p* < 0.01 versus SCI+sh-NC group.

We evaluated the effect of Atf3 knockdown on SCI by administering shAtf3 to a rat model of SCI. We observed significantly elevated mRNA and protein expression of Atf3 in SCI rats, whereas treatment with shAtf3 effectively reduced Atf3 levels in the spinal cord tissue (Figure 6). Histological staining demonstrated that Atf3 knockdown alleviated tissue damage (Figure 6) and restored collagen deposition (Figure 6) and the number of Nissl bodies (Figure 6) in spinal cord tissue. We further observed that antioxidant factors, including Nrf2, NQO-1, and HO-1, were notably reduced in SCI rats, but their expression recovered following shAtf3 treatment (Figure 6). Moreover, shAtf3 treatment lowered the levels of oxidative enzymes, such as MPO and MDA, while increasing the levels of the antioxidants GSH and SOD (Figure 6).

**Figure 7:**
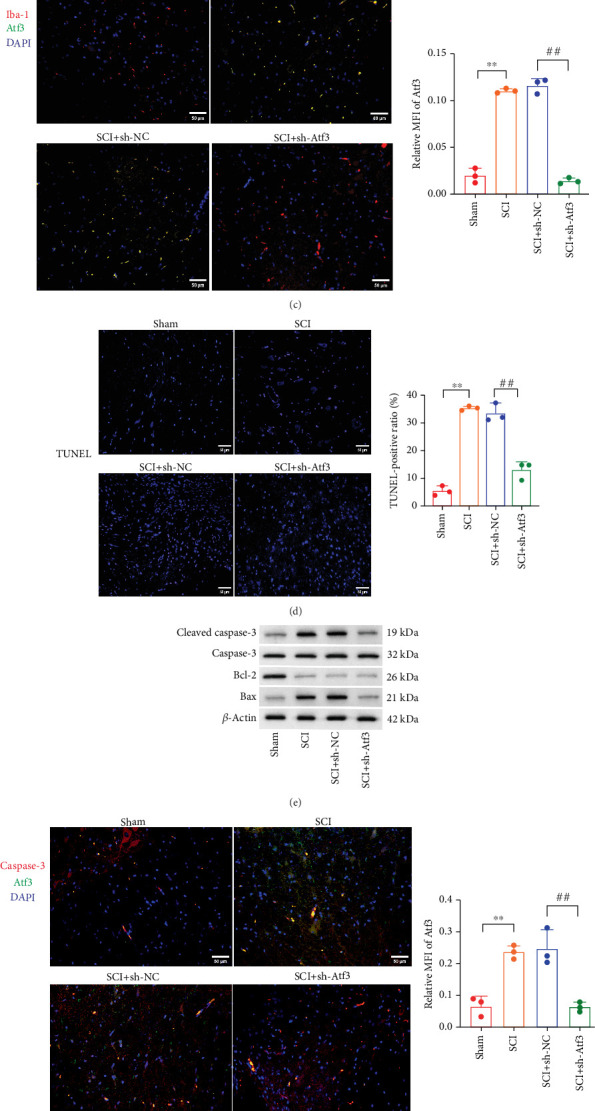
Atf3 mainly colocalizes with neuronal cells, and knockdown of Atf3 attenuates the inflammatory response and apoptosis. (a–c) Colocalization of Atf3 and NeuN, GFAP, and Iba-1 was analyzed by immunofluorescence in the spinal cord tissues of the Sham, SCI, SCI + sh-NC, and SCI + sh-Atf3 groups (scale bar = 50* μ*m). (d) Detection of apoptosis in spinal cord tissues of each group by TUNEL staining (scale bar = 50* μ*m). (e) Expression of apoptotic proteins (cleaved Caspase-3, Caspase-3, Bax, and Bcl-2) was analyzed by Western blot in each group. (f) The expression of Atf3 and Caspase-3 in spinal cord tissues of each group was analyzed by immunofluorescence staining (scale bar = 50* μ*m). (g) The mRNA levels of inflammatory cytokines were analyzed by qPCR in each group. (h) Secretion levels of inflammatory cytokines in each group were analyzed by ELISA. (i) Protein levels of inflammatory cytokines in each group were analyzed by Western blot. Data are presented as mean ± SD, *n* = 3. ∗*p* < 0.05, ∗∗*p* < 0.01 versus Sham group; ##*p* < 0.01 versus SCI + sh-NC group.

Additionally, Atf3 was predominantly localized in neurons, as shown by its colocalization with NeuN, with lower expression in astrocytes and microglia, as indicated by GFAP and Iba-1 markers (Figures 7a, 7b, and 7c) suggesting that Atf3 acts primarily on neurons rather than glial cells. Treatment with shAtf3 also reduced the number of TUNEL-positive cells, indicating alleviation of cell apoptosis (Figure 7d). The results of Western blot assays further indicated and immunofluorescence staining that Atf3 knockdown downregulated the levels of proapoptotic proteins, specifically cleaved caspase-3 and Bax (Figure 7e,f). Furthermore, the results of qPCR, ELISA, and Western blot demonstrated that knockdown of Atf3 suppressed the expression and secretion of the proinflammatory cytokines TNF-*α*, IL-1*β*, and IL-6 (Figures 7g, 7h, and 7i).

### 3.7. Knockdown of Atf3 Alleviates OS, Inflammatory Response, and Neuronal Death In Vitro

**Figure 8:**
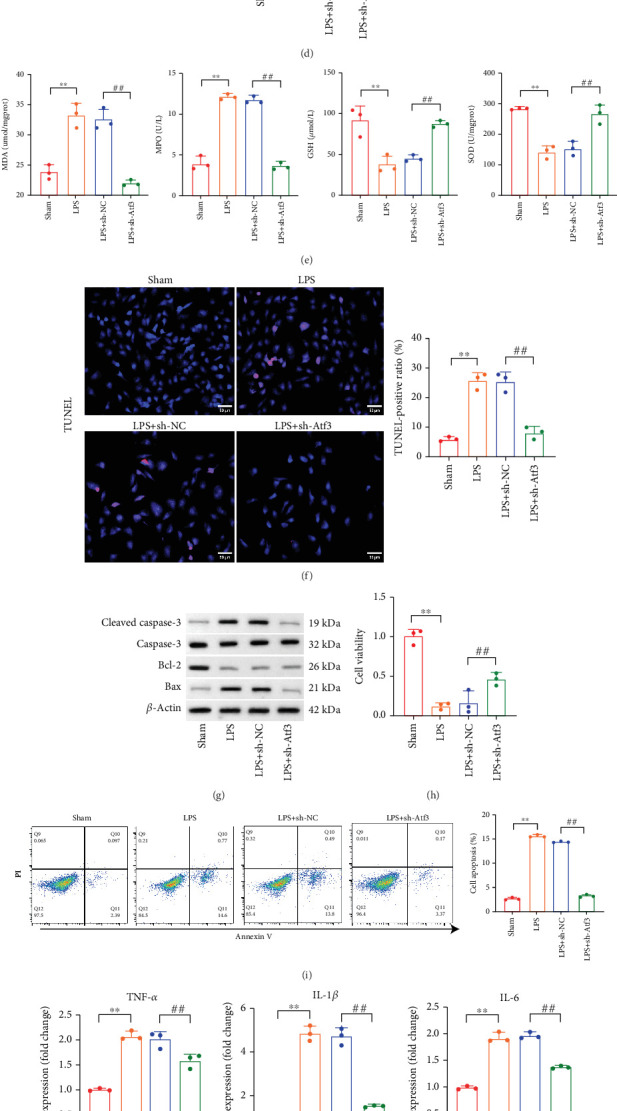
Knockdown of Atf3 attenuates oxidative stress, inflammatory response, and neuronal apoptosis in vitro. SCI cell model was constructed by stimulating PC12 cells with LPS. The (a) mRNA and (b) protein levels of Atf3 and the (c) mRNA and (d) protein levels of antioxidant factors (Nrf2, NQO-1, and HO-1) were detected in Sham, LPS, LPS + sh-NC, and LPS + sh-Atf3 groups by qPCR and Western blot. (e) The levels of oxidative enzymes (MDA and MPO) and antioxidant enzymes (GSH and SOD) were detected in each group. (f) Detection of apoptosis in each group by TUNEL staining (scale bar = 50* μ*m). (g) Expression of apoptotic proteins (cleaved Caspase-3, Caspase-3, Bax, and Bcl-2) in each group was analyzed by Western blot. (h) Cell viability of each group was analyzed by CCK-8. (i) Cell apoptosis was analyzed by flow cytometry in each group. (j) Detection of mRNA levels of inflammatory cytokines in each group by qPCR. (k) The secretion of inflammatory cytokines in each group was analyzed by ELISA. (l) Protein levels of inflammatory cytokines in each group were analyzed by Western blot. Data are presented as mean ± SD, *n* = 3. ∗∗*p* < 0.01 versus Sham group; ^##^*p* < 0.01 versus LPS + sh-NC group.

We established an LPS-stimulated cell model to investigate the effects of Atf3 knockdown in vitro. Consistent with the results in vivo, LPS stimulation induced Atf3 expression, which was suppressed by shAtf3 (Figure 8). The mRNA and protein levels of the antioxidative factors Nrf2, NQO-1, and HO-1, which were repressed by LPS, were notably restored by shAtf3 (Figure 8). Additionally, shAtf3 treatment decreased the levels of the oxidative enzyme MPO and the products of MDA while increasing the levels of the antioxidants GSH and SOD (Figure 8). The number of TUNEL-positive cells reduced following shAtf3 treatment, indicating a decrease in apoptosis (Figure 8). The results of the Western blot assay further indicated that Atf3 knockdown downregulated proapoptotic proteins, specifically cleaved Caspase-3 and Bax (Figure 8). CCK-8 and flow cytometry results confirmed that Atf3 knockdown restored neuronal viability (Figure 8) and reduced apoptosis (Figure 8). The LPS-induced expression and secretion of the proinflammatory cytokines TNF-*α*, IL-1*β*, and IL-6 were also suppressed by shAtf3 (Figures 8, 8, and 8).

### 3.8. Atf3 Regulates the NF-*κ*B Signaling in Neurons

**Figure 9:**
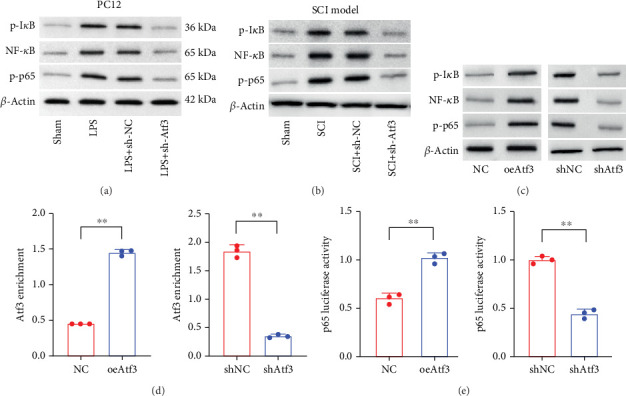
Atf3 regulates NF-*κ*B signaling in neuronal cells. Western blot detection of NF-*κ*B/p65 signaling pathway in (a) LPS-induced PC12 cells and (b) SCI model. (c) Western blot detection of NF-*κ*B/p65 cascade in Atf3 overexpression vector or shAtf3-treated PC12 cells. (d) ChIP-qPCR of Atf3 binding to p65 promoter. (e) Luciferase reporter gene assay to determine p65 promoter activity. Data are presented as mean ± SD, *n* = 3. ∗∗*p* < 0.01 versus NC/shNC group.

We observed activation of the NF-*κ*B/p65 signaling cascade in LPS-induced PC12 cells (Figure 9a) and spinal cord tissue of SCI rats (Figure 9b), which was repressed following Atf3 knockdown. Furthermore, overexpression of Atf3 increased NF-*κ*B/p65 activation, while its knockdown reduced it (Figure 9c). Results from the ChIP-qPCR assay indicated that Atf3 overexpression and knockdown enhanced and reduced the binding of Atf3 to the p65 promoter, respectively (Figure 9d). Additionally, the results of the luciferase reporter gene assay suggested that Atf3 promoted p65 promoter activity, whereas shAtf3 was inhibited (Figure 9e).

### 3.9. Atf3 Alleviates OS, Inflammatory Response, and Neuronal Death via NF-*κ*B Regulation

**Figure 10:**
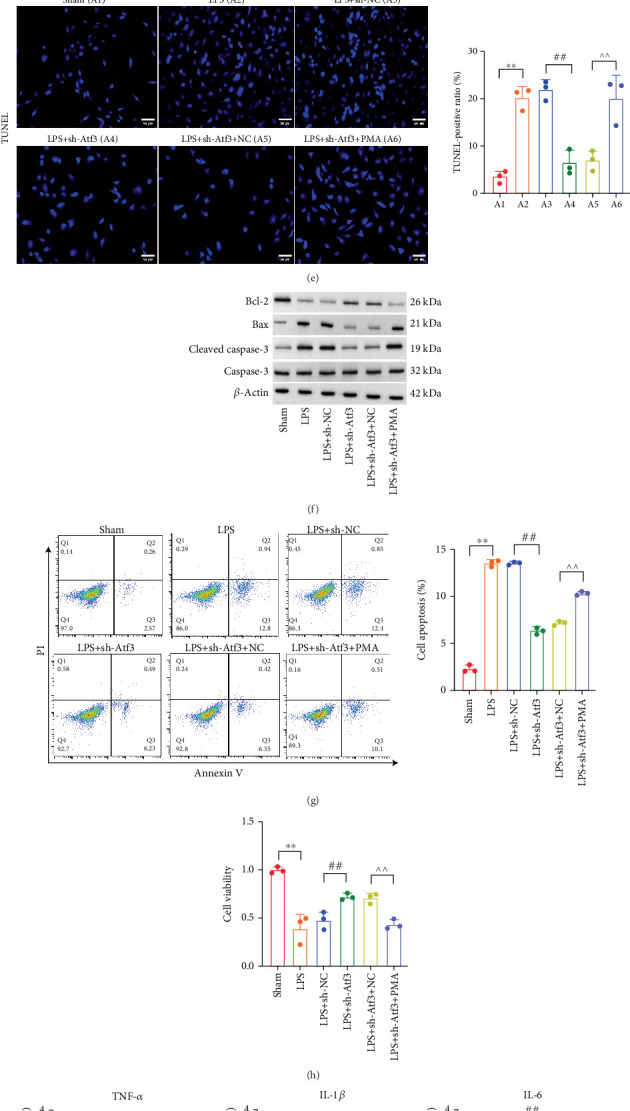
Atf3 affects oxidative stress, inflammatory response, and neuronal apoptosis in vitro by regulating NF-*κ*B. (a) Protein expression of the NF-*κ*B signaling pathway in PC12 cells was analyzed by Western blot. Detection of (b) mRNA and (c) protein levels of antioxidant factors (Nrf2, NQO-1, and HO-1) in Sham, LPS, LPS + sh-NC, LPS + sh-Atf3, LPS + sh-Atf3 + NC, and LPS + sh-Atf3 + PMA groups of PC12 cells by qPCR and Western blot levels. (d) Levels of oxidative enzymes (MDA and MPO) and antioxidant enzymes (GSH and SOD) in each group of PC12 cells. (e) Detection of apoptosis in each group by TUNEL staining (scale bar = 50* μ*m). (f) The expression levels of apoptotic proteins (cleaved Caspase-3, Caspase-3, Bax, and Bcl-2) in each group were analyzed by Western blot. (g) Detection of apoptosis in each group by flow cytometry. (h) Cell viability was analyzed by CCK-8 in each group. (i) mRNA levels of inflammatory cytokines in each group were analyzed by qPCR. (j) The secretion level of inflammatory cytokines in each group was analyzed by ELISA. (k) Protein expression levels of inflammatory cytokines in each group were analyzed by Western blot. Data are presented as mean ± SD, *n* = 3. ∗∗*p* < 0.01 versus Sham group; ^##^*p* < 0.01 versus (LPS + sh-NC) group; ^∧∧^*p* < 0.01 versus LPS + sh-Atf3 + NC group.

To further explore the role of NF-*κ*B in Atf3-mediated effects, we administered the NF-*κ*B activator PMA in an LPS-induced PC12 model. The NF-*κ*B activator restored NF-*κ*B signaling, which was suppressed by shAtf3 (Figure 10a). Activation of NF-*κ*B reversed shAtf3-induced upregulation of the antioxidative factors Nrf2, NQO-1, and HO-1 (Figure 10b,c). NF-*κ*B activation also restored the levels of the oxidative enzyme MPO and the products of MDA while reducing the levels of antioxidant GSH and SOD, counteracting the effects of shAtf3 (Figure 10d). Moreover, shAtf3 treatment reduced the number of TUNEL-positive cells (Figure 10e), downregulated the levels of proapoptotic proteins cleaved caspase-3 and Bax (Figure 10f), repressed apoptosis in PC12 cells (Figure 10g), and promoted cell proliferation (Figure 10h). These protective effects of shAtf3, however, were reversed by NF-*κ*B activation. Moreover, the LPS-induced expression and secretion of the proinflammatory cytokines TNF-*α*, IL-1*β*, and IL-6 were suppressed by shAtf3 and recovered by the NF-*κ*B activator (Figures 10i, 10j, and 10k).

### 3.10. Atf3 Modulates Spinal Cord Injury, OS, and Inflammatory Response via NF-*κ*B Regulation

**Figure 11:**
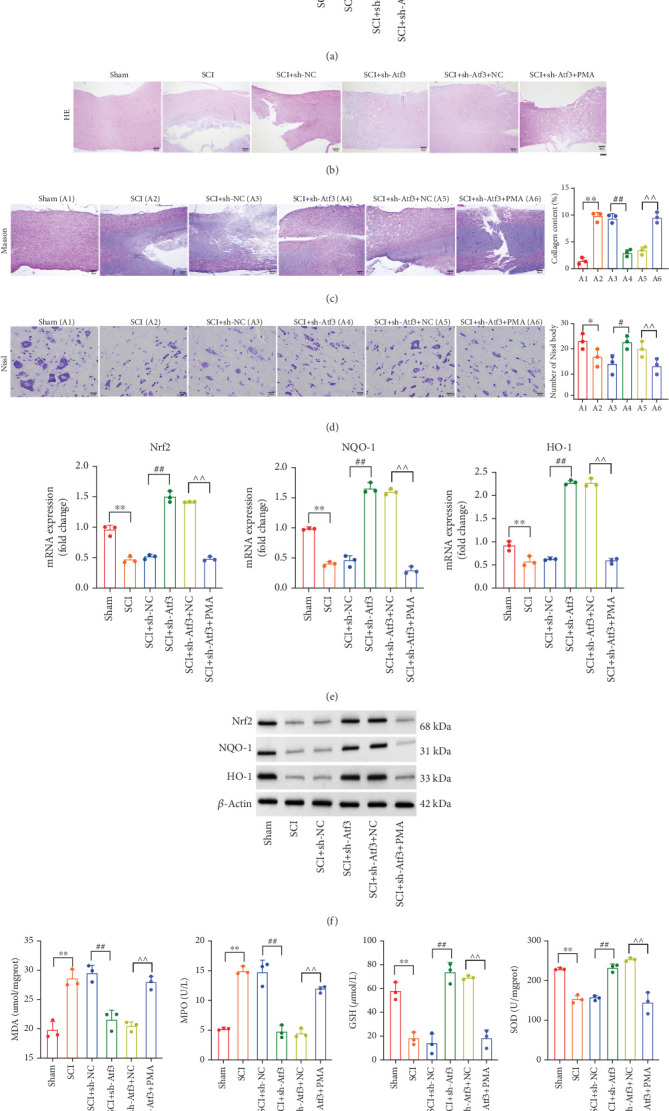
Atf3 affects SCI and oxidative stress by regulating NF-*κ*B. (a) Protein levels of NF-*κ*B signaling cascade in Sham, SCI, SCI + sh-NC, SCI + sh-Atf3, SCI + sh-Atf3 + NC, and SCI + sh-Atf3 + PMA groups were analyzed by Western blot. Tissue damage in each group by (b) HE staining, (c) Masson staining, and (d) Nissl staining (scale bar = 50* μ*m). (e) mRNA and (f) protein levels of antioxidant factors (Nrf2, NQO-1, and HO-1) in spinal cord tissues of each group were analyzed by qPCR and Western blot. (g) Expression levels of oxidative enzymes (MDA and MPO) and antioxidant enzymes (GSH and SOD) in spinal cord tissues of each group. Data are presented as mean ± SD, *n* = 3. ∗*p* < 0.05, ∗∗*p* < 0.01 versus Sham group; ^#^*p* < 0.05, ^##^*p* < 0.01 versus (SCI + sh-NC) group; ^∧∧^*p* < 0.01 versus SCI + sh-Atf3 + NC group.

**Figure 12:**
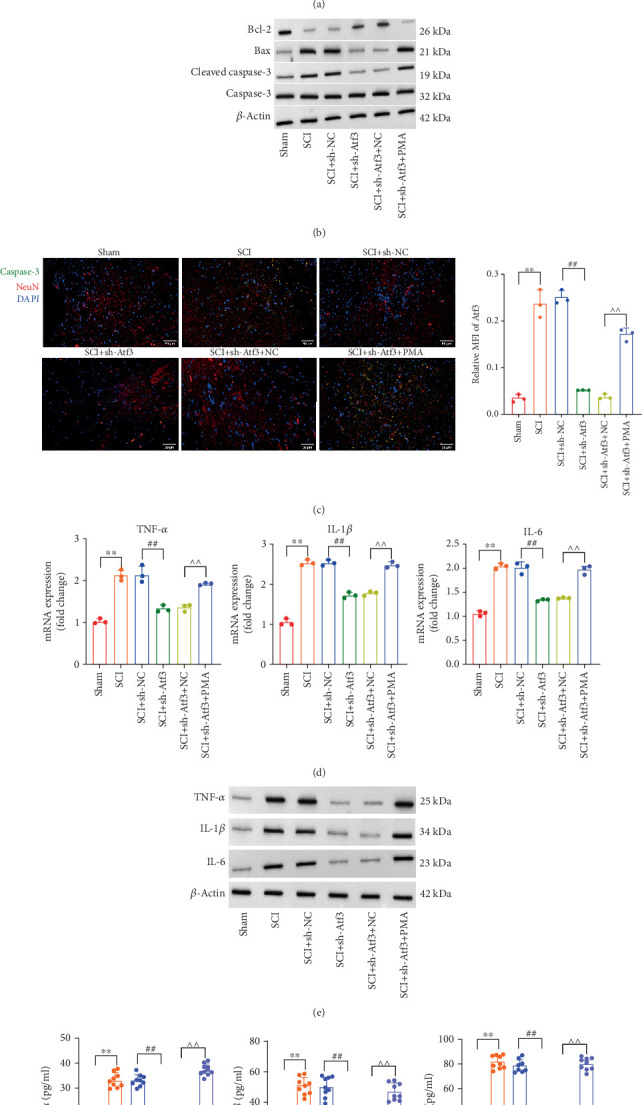
Atf3 affects inflammatory response and neuronal apoptosis by regulating NF-*κ*B. (a) Neuronal cell apoptosis was analyzed by TUNEL staining in spinal cord tissues of Sham, SCI, SCI + sh-NC, SCI + sh-Atf3, SCI + sh-Atf3 + NC, and SCI + sh-Atf3 + PMA groups (scale bar = 50* μ*m). (b) The expression levels of apoptotic proteins (cleaved Caspase-3, Caspase-3, Bax, and Bcl-2) in each group were analyzed by Western blot. (c) The expression levels of NeuN and Caspase-3 in spinal cord tissues of each group were analyzed by immunofluorescence staining (scale bar = 50* μ*m). (d) mRNA levels of inflammatory cytokines were analyzed by qPCR in each group. (e) Protein levels of inflammatory cytokines in each group were analyzed by Western blot. (f) Secretion levels of inflammatory cytokines in each group were determined by ELISA. Data are presented as mean ± SD, *n* = 3. ∗∗*p* < 0.01 versus Sham group; ^##^*p* < 0.01 versus (SCI + sh-NC) group; ^∧∧^*p* < 0.01 versus SCI + sh-Atf3 + NC group.

Subsequently, we examined the effects of NF-*κ*B signaling on Atf3-regulated SCI. Treatment with the NF-*κ*B activator recovered shAtf3-repressed NF-*κ*B signaling in spinal cord tissue **(**Figure 11**)**. Histological analysis showed that NF-*κ*B activation induced tissue damage and reduced collagen deposition and the number of Nissl bodies in spinal cord tissue, which abolished the protective effects of shAtf3 **(**Figures 11, 11, and 11**)**. Furthermore, the NF-*κ*B activator suppressed shAtf3-mediated recovery of the antioxidative factors Nrf2, NQO-1, and HO-1 at both the mRNA and protein levels (Figure 11). The reduction in oxidative enzymes MPO and the products of MDA, along with the elevated levels of antioxidants GSH and SOD achieved by shAtf3, was also reversed by NF-*κ*B activation (Figure 11). The results of the TUNEL assay and Western blot indicated that NF-*κ*B activation induced neuronal apoptosis, counteracting the antiapoptotic effects of shAtf3 (Figures 12, 12, and 12). Additionally, the results of qPCR, Western blot, and ELISA revealed that NF-*κ*B activation restored the levels of the proinflammatory cytokines TNF-*α*, IL-1*β*, and IL-6, which were repressed by shAtf3 (Figures 12, 12, and 12).

## 4. Discussion

To identify key regulators of OS in SCI, we conducted bioinformatics analyses that pinpointed Atf3 and the NF-*κ*B pathway as central players. Previous studies report that Atf3, a neuronal injury marker in the nervous system, induces OS and impairs CNS regenerative capacity, thereby hindering neurological recovery [12, 26]. Building on these findings, we employed lentiviral vectors to knock down Atf3 in vivo and in vitro, utilizing LPS-induced PC12 cell and rat SCI models. Our results demonstrated that Atf3 exacerbates neuronal damage and SCI progression by amplifying OS and inflammation via the NF-*κ*B pathway. These findings position Atf3 as a critical mediator of SCI-associated oxidative-inflammatory pathology.

As a stress-inducible transcription factor, Atf3 maintains stable expression under physiological conditions but becomes dysregulated in pathologies involving inflammation, oxidative and ER stress, and cell death [11, 27]. Under stress, Atf3 acts as a metabolic-immune interface regulator, preserving cellular homeostasis. Fifteen post-SCI, Atf3 expression surges at 4 h, peaks at 24 h (plateau phase), and declines by Day 7 [12, 13, 28, 29]. While Atf3 promotes axonal regeneration across species—from lampreys to mammals—its neuroprotective effects in zebrafish SCI models via inflammation modulation contrast with its role in exacerbating mitochondrial dysfunction and OS in other contexts [30, 31]. Our data confirm low baseline Atf3 expression in healthy spinal neurons, with pathological upregulation correlating with heightened oxidative/inflammatory markers. Atf3 knockdown reversed these effects, underscoring its regulatory potential. Notably, immunofluorescence revealed predominant Atf3 colocalization with neurons (NeuN^+^) rather than microglia (Iba-1^+^) or astrocytes (GFAP^+^) in SCI models, aligning with neurons' central role in post-SCI motor recovery [32].

The role of Atf3 appears to be complex. Atf3 is significantly upregulated in spinal motor and sensory neuron expression after nerve injury, contributing to neuronal regeneration, and is regarded as a RAG [12]. However, it has also been shown that Atf3 overexpression aggravates the level of cellular OS and mitochondrial dysfunction, and may modulate inflammatory responses by controlling the expression of multiple cytokines and chemokines and thus influence disease onset and progression [33]. This dual role of Atf3 as an important OS progenitor may be dependent on the heterogeneity of Atf3 related to different microenvironments, different signaling pathways, and disease development. By constructing a rat SCI model and experimenting with an in vitro LPS-induced SCI model in PC12 cells, we observed that Atf3 expression was upregulated after spinal cord injury, which led to tissue damage, OS, and inflammation in spinal cord tissues. Knockdown of Atf3 decreased the levels of OS markers (MPO and MDA), increased antioxidant responses (GSH and SOD), and improved histological outcomes. This suggests that Atf3 knockdown attenuates secondary injury by restoring oxidative homeostasis, which is critical for protecting neurons from damage in the postinjury environment. Our study suggests that Atf3 enhances OS and inflammatory responses after SCI, highlighting its environment-dependent function in the pathophysiology of SCI, which warrants further investigation.

Studies have shown that Atf3 exerts an inflammatory inhibitory effect by modulating the NF-*κ*B pathway [34, 35]. In SCI treatment, tomatidine alleviates OS and inflammation by inhibiting the NF-*κ*B signaling pathway [36]. Similarly, phillygenin inhibits neuroinflammation and facilitates functional recovery after spinal cord injury by inhibiting the NF-*κ*B signaling pathway via TLR4 [37]. The NF-*κ*B signaling pathway is also inhibited by phillygenin. The NF-*κ*B signaling pathway, as a key regulatory hub in the CNS, plays a molecular switching role in the regulation of synaptic dynamic plasticity, memory formation, and amplification of the inflammatory cascade of neurological injury [17, 38]. Astrocytes can respond to external stimuli by activating the intracellular NF-*κ*B signaling pathway, leading to the production of the proinflammatory factor IL-6 by astrocytes after SCI. IL-6 exacerbates the inflammatory response and affects the functional recovery of SCI [18]. It has also been shown that ROS and NF-*κ*B signaling pathways interact, proposing that NF-*κ*B has a regulatory role on the level of ROS in cells [19, 20]. Despite the overwhelming evidence that Atf3 exerts a bidirectional effect through signaling pathways such as NF-*κ*B, it is not clear what the regulatory mechanism of Atf3 is in SCI. We demonstrated that the knockdown of Atf3 inhibited OS and inflammation levels in vivo and in vitro by activating the NF-*κ*B signaling pathway in SCI rats and PC12 cells, while the activation of NF-*κ*B partially reversed the relevant functions and cell viability. It has been shown that Atf3 regulates the expression of OS-related genes, such as antioxidant factors (Nrf2, NQO-1, and HO-1), oxidative enzymes (MPO, and MDA), and antioxidant enzymes (GSH, and SOD), which enhances cellular antioxidant capacity and reduces cellular damage by ROS, which may be mediated through the regulation of the NF-*κ*B signaling pathway that affects antioxidant gene expression to produce effects [39, 40]. Our results further suggest that Atf3 may regulate neuronal OS and inflammatory responses through NF-*κ*B and thus affect SCI progression, indicating that the functional inhibition of Atf3 as a key transcriptional regulatory node in the oxidative-inflammatory-apoptotic cascade after SCI significantly ameliorates secondary neurological injury. These findings provide new perspectives for further understanding of molecular mechanisms in SCI.

In recent years, it has been shown that the NF-*κ*B signaling pathway, as a key regulatory hub within the CNS, plays a molecular switching role in the modulation of synaptic dynamic plasticity, memory formation, and amplification of inflammatory cascades of nerve injury [38, 41]. It has been shown in the literature that Atf3 can regulate NF-*κ*B transcription by altering the phosphorylation of I*κ*B*α*, and that the synergistic effect of Atf3 and NF-*κ*B synergistically regulates cellular OS, thus jointly participating in the regulation of genes related to cell survival or apoptosis [42]. It has also been shown that there is also an interaction between the ROS and NF-*κ*B signaling pathways, and it is proposed that NF-*κ*B has a modulatory effect on the level of ROS in cells [19, 20]. To further clarify the mechanism by which Atf3 regulates OS and inflammatory responses, we observed that overexpression of Atf3 enhanced NF-*κ*B/p65 signaling, whereas knockdown of Atf3 inhibited this effect. This regulation of NF-*κ*B by Atf3 was further supported by the ChIP-qPCR assay, which showed that Atf3 binds directly to the NF-*κ*B p65 promoter, which suggests that Atf3 acts upstream of NF-*κ*B in SCI, possibly through direct transcriptional regulation, and modulates the inflammatory and oxidative responses that exacerbate spinal cord injury. Although the relationship between Atf3 and the NF-*κ*B pathway in the pathophysiology of SCI has not been fully elucidated, our results suggest that Atf3 promotes post-SCI OS and inflammation via the NF-*κ*B signaling pathway, thus establishing a critical mechanistic link between these two important signaling molecules.

Despite the significance of our findings, there are still some limitations of this study that should be considered. First, we experimentally observed Atf3 changes in animals and cells for 24 h of injury, mainly based on a large number of previous reports in the literature that Atf3 begins to rise at 4 h after SCI, peaks at 24 h, and then enters a plateau period, with a decline beginning on day 7. Therefore, we chose to observe for 24 h, but of course, a longer period of time would certainly be meaningful for the study, and as a next step, we will conduct a longer-term observation. Second, SCI functional recovery largely depends on spinal neurons, and post-SCI Atf3 expression is concentrated in neurons with minimal glial expression. When we conducted in vivo studies, we assessed Atf3 in the whole spinal cord tissue and did not assess the role of Atf3 on glial cells, which lacked certain cell specificity. The next step is that we will carry out immunofluorescence double-labeling or specific knockdown of Atf3 on neuronal cells, to more clearly define the expression and role of Atf3 on neuronal cells. Third, we also found low expression of Atf3 on glial cells in rat spinal cord tissues after SCI. Whether the progression of the inflammatory response after SCI is regulated by Atf3 after modulation of neuronal cells through paracrine, exosomes, etc., which affects glial cells, or whether the rapid and high expression of Atf3 on glial cells is in the process of modulation needs to be further explored. Fourth, although shAtf3 treatment was effective in reducing Atf3 levels, further studies are needed to investigate the potential off-target effects, the feasibility of knocking down Atf3 in animals, and the cell type-specific responses. Fifth, we analyzed Atf3 by bioinformatics and found that it may affect OS and inflammation through the NF-*κ*B signaling pathway, but the signaling pathway dependence and whether it is transient/sustained activated have not been further investigated and should be further explored in future studies. Sixth, Atf3 affects OS and inflammation by regulating the NF-*κ*B signaling pathway. The development of inhibitors (e.g., small molecule compounds, gene editing technology) has potential, but it faces the challenges of CNS delivery (blood-brain/spinal cord barrier penetration) and safety risks (off-target effects, interference with physiological repair function). Nanocarrier-targeted delivery, local intrathecal administration, and conditional gene modulation are needed, as well as the impact of individual differences (trace element levels, genetic polymorphisms) on efficacy. In the future, we need to combine dynamic regulatory strategies, multigroup biomarker development, and interdisciplinary collaborations to promote the role of Atf3-related drugs in the CNS.

In conclusion, our study identified Atf3 as a key regulator of OS and inflammation in SCI by regulating NF-*κ*B signaling. By elucidating this regulatory mechanism, our results have improved our understanding of OS and neuroinflammatory processes in SCI and identified Atf3 as a critical gene in regulating the pathological processes of OS and inflammation in SCI. Future studies on the complex interactions between Atf3, OS, and inflammation will not only deepen our understanding of the mechanism but also provide an experimental basis for mitigating secondary SCI pathology.

## 5. Conclusion

Downregulation of Atf3 mitigates OS and inflammation in SCI, potentially by regulating neuronal biological functions via the NF-*κ*B pathway. These findings provide a theoretical foundation for understanding SCI pathogenesis and identifying therapeutic targets.

## Data Availability

The raw data used to support the findings of this study are available from the corresponding authors upon request.

## Supporting Information

Supporting Information Additional supporting information can be found online in the Supporting Information section. Figures S1 and S2 and Tables S1 and S2 provide additional data supporting the main findings, including validation of shAtf3 specificity (Supporting Information 1 and Table S1), all relevant primer sequences in this study (Table S2) and raw data for CHIP-qPCR in Figure 9 (Figure S1 and Figure S2).
