# Supplementary material for: Atf3 Promotes Spinal Cord Injury by Exacerbating Neuronal Oxidative Stress and Inflammation via the NF-κB Signaling Pathway
Source: Int J Genomics. 2025 Aug 11;2025:1027388. doi: 10.1155/ijog/1027388 (PMC12360882; doi:10.1155/ijog/1027388)
Supplement: Supporting Information — Additional supporting information can be found online in the Supporting Information section. Figures S1 and S2 and Tables S1 and S2 provide additional data supporting the main findings, including validation of shAtf3 specificity (Supporting Information 1 and Table S1), all relevant primer sequences in this study (Table S2) and raw data for CHIP-qPCR in Figure 9 (Figure S1 and Figure S2). [file 1027388.f1.docx]

**Supplementary Material**

**Supplementary Material 1:** **Validation of shAtf3 specificity**

A lentiviral vector (pCDH-CMV-MCS-EF1-puro) was synthesized. Three shRNA interference targets (shAtf3-1,shAtf3-2,shAtf3-3) were designed against the Atf3 gene. The shRNA sequences and corresponding viral titers (measured in TU/mL) were as follows (Table S1). PC-12 cells were transfected with the lentivirus, and Atf3 expression was analyzed by qPCR post-transfection. qPCR results demonstrated that shAtf3-3 most effectively knocked down Atf3 (p < 0.001 vs. control), reducing its expression to 30% of baseline levels. Based on this efficiency, shAtf3-3 was selected for subsequent in vivo experiments.

**Table S1: shRNA interference target sequences and corresponding viral titers**

| Carrier name | Insertion sequence | Viral titers |
| --- | --- | --- |
| pCDH-CMV-MCS-EF1-puro-si-ATF3-1 | UAGAAGUGCACAGGAAGCCAG | 6.32×10^8^ |
| pCDH-CMV-MCS-EF1-puro-si-ATF3-2 | UCAGGUCUGAUGAAACUCCCG | 5.48×10^8^ |
| pCDH-CMV-MCS-EF1-puro-si-ATF3-3 | UUGACAGGCUAGGAAUACUGG | 8.64×10^8^ |

**Table S2: All relevant primer sequences in this study**

| Primer name | Primer sequence |
| --- | --- |
| R-Nrf2-F | CAGGACAGAAGCTGTGCATC |
| R-Nrf2-R | CTTCGTGGAAGAAGGCATGG |
| R-NQO-1-F | TTCTGTGGCTTCCAGGTCTT |
| R-NQO-1-R | TTCTTCCACCCTTCCAGGAC |
| R-HO-1-F | GAATTCAGCATGCCCCAGGATTTG |
| R-HO-1-R | TCTAGACTAGCTGGATGTTGAGCAGGA |
| R-TNF-α-F | CAGCCTCTTCTCATTCCTGCTCG |
| R-TNF-α-R | GTCTGGGCCATGGAACTGATGAG |
| R-IL-1β-F | AGTTTGAGTCTGCACAGTTCCCC |
| R-IL-1β-R | ACGGGTTCCATGGTGAAGTCAAC |
| R-IL-6-F | GCCACTGCCTTCCCTACTTCAC |
| R-IL-6-R | CTGGTCTGTTGTGGGTGGTATCC |
| R-NF-κB-R | CACCCGGCTCACAGAATTTC |
| R-NF-κB-R | GCCCCAACAGTCGACCTTTA |
| R-GAPDH-F | GCCCAGAACATCATCCCTGCAT |
| R-GAPDH-R | GCCTGCTTCACCACCTTCTTGA |
| R-Atf3-F | ATTCGCCATCCAGAACAAGC |
| R-Atf3-R | CCACCTCAGACTTGGTGACT |

**Supplementary Material 2: Raw data for CHIP-qPCR in F4D**

ChIP-qPCR showed that overexpression (Figure S1) and knockdown (Figure S2) of Atf3 enhanced and reduced the binding of Atf3 to p65, respectively.

**Figure S1: Amplification and Melting curves of Atf3 (NC vs oeAtf3)**

**
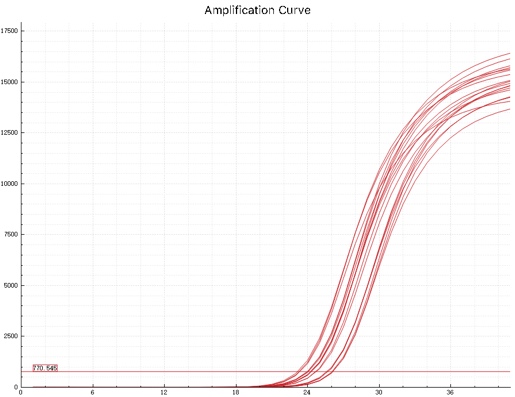

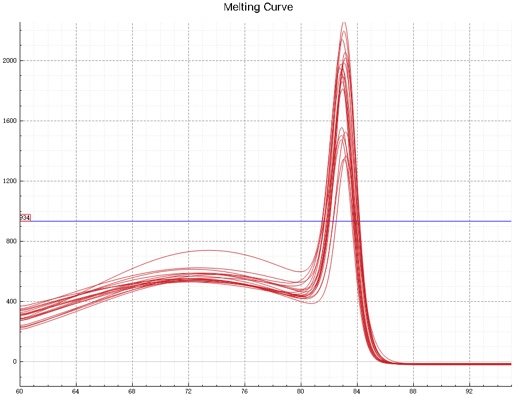
**

**Figure S2: Amplification and Melting curves of Atf3(shNC vs shAtf3)**

**
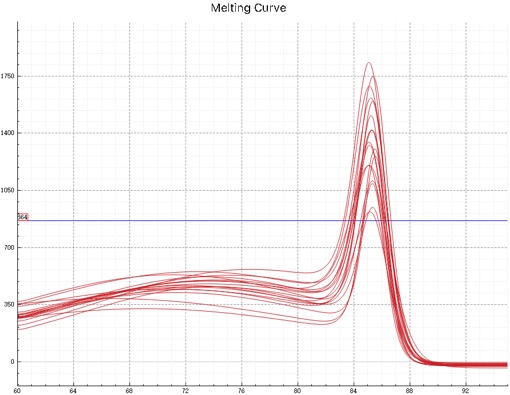

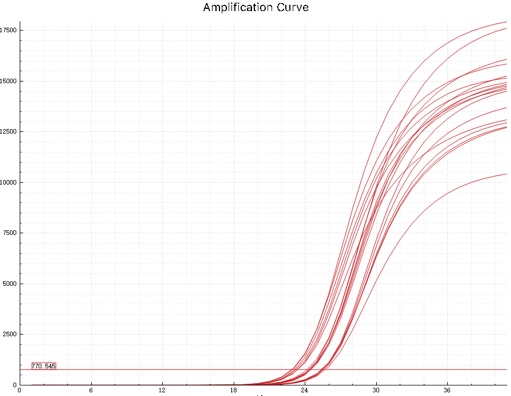
**
